# The effect of Tributyltin on thyroid follicular cells of adult male albino rats and the possible protective role of green tea: a toxicological, histological and biochemical study

**DOI:** 10.1186/s41935-017-0012-z

**Published:** 2017-07-18

**Authors:** Fatma M. M. Badr El Dine, Iman M. Nabil, Fatma I. Dwedar

**Affiliations:** 1 Departments of Forensic Medicine and Clinical Toxicology, Faculty of Medicine, Alexandria University, Champollion Street, El- Khartoum Square, Azarita Medical Campus, Alexandria, Egypt; 2 Histology and cell biology, Faculty of Medicine, Alexandria University, Alexandria, Egypt; 3 Medical Biochemistry, Faculty of Medicine, Alexandria University, Alexandria, Egypt

**Keywords:** Tributyltin, Organotin, Green tea extract, Thyroid

## Abstract

**Introduction:**

Tributyltin is one of the important and wide-spread persistent organic contaminants that accumulate in the food chain. It is suspected to cause endocrine-disrupting effects in mammals, due in part to its possible transfer through marine food chains and to the consumption of contaminated seafood.

**Aim of the work:**

Was to study the possible toxic effect of Tributyltin on thyroid follicular cells of adult male albino rats and to evaluate the possible protective role of green tea.

**Material and methods:**

Forty-five adult male albino rats were included and randomly divided into 3 equal groups: a control group (Group I); Group II: received tributyltin chloride (TBT) dissolved in corn oil orally in a dose of 5 mg/kg for 30 days. Group III: received tributyltin chloride in the same dose with concomitant oral administration of green tea extract for 30 days. At the end of the experiment, the animals were sacrificed and blood samples were subjected to hormonal assay for T3, T4 and TSH levels. Malondialdehyde and reduced glutathione were assessed. The thyroid tissue was processed for histological and ultrastructure examination. The colloid area of thyroid follicles was evaluated morphometrically and statistically analyzed.

**Results:**

A significant decrease in T3 and T4 levels and serum reduced glutathione in the group II when compared with the other groups. Furthermore, a significant increase in serum Malondialdehyde and TSH levels was recorded in group II treated group by comparison to the other two groups. Histopathological and ultrastructural changes of thyroid gland follicles were detected in tributyltin treated rats; the follicular cells appeared swollen and vacuolated. Epithelial stratification was noticed in some foci with excessive vacuolation of the colloid. Dilated rough endoplasmic reticulum filled with flocculent material and increased number of lysosomes were also detected together with variation in shape and size of the nuclei. A marked improvement in the histological features of thyroid follicles was noticed in group III.

**Conclusion:**

Tributyltin induces oxidative stress in rats as well as anti-thyroid effect. The green tea extract is useful in combating tissue injury that is a result of tributyltin toxicity.

## Introduction

Exposure to endocrine disrupting chemicals grabs more attention nowadays and represents international concern of many national and international health organizations, as well as being a political issue in various countries (Acerini & Hughes, 2006).

According to the World Health Organization (WHO), an endocrine disruptor is defined as “…an exogenous substance or mixture that modulates function(s) of the endocrine system and consequently causes adverse health effects in an intact organism, or its progeny, or (sub)populations” (Damstra et al., 2002).

Organotin compounds (OT), as tributyltin (TBT) and triphenyltin (TPT), are prevalent contaminants that have been widely used as biocides, agriculture fungicides and wood preservatives. Furthermore, they have been utilized as disinfecting agents in circulating industrial cooling waters, as well as, antifouling paints for marine vessels (Antizar-Ladislao, 2008).

Even though the usage of Tributyltins has been banned since 2008, they continue to persist in the environment, leading to severe contamination of the ecosystems as well as its accumulation in biological tissues (Horiguchi, 2012).

TBT at relatively high concentrations as hundreds of nanomoles has been found in human blood (Antizar-Ladislao, 2008) and the lipophilicity of organotin compounds favors their toxicity at membrane level, as well as disrupting diverse biological processes, involved in the endocrine, (Sharan et al., 2013) the immune, (Brown & Whalen, 2015) and nervous systems (Dong et al., 2006).

Moreover, TBT are reported to be a perilous factor for cardiovascular disease impairing the coronary vascular reactivity to estradiol, (dos Santos et al., 2012) as well as, altering aorta morphology and functionality (Rodrigues et al., 2014).

Accumulating data suggests that TBT can act as an endocrine disruptor. TBT has been known to produce imposex (a superimposition of male features in females) in marine gastropods through inhibition of aromatase enzyme that converts androgen to estrogen (Sousa et al., 2009; Lima et al., 2011). In mammals, nanomolar concentrations of tributyltin disturbed steroidogenesis (Yamazaki et al., 2005). Organotin compounds as endocrine disruptors are indicated as a possible cause of congenital hypothyroidism (Adeeko et al., 2003).

Recently, Tributyltin toxicity is considered as a precipitating factor for transgenerational obesity by disturbing the levels of key hormones linked to energy homeostasis (Nicole, 2013; Chamorro-García et al., 2013).

TBT has also been reported to be an environmental risk factor for Parkinson’s disease, by inhibiting dopamine biosynthesis and enhancing L-DOPA-induced cytotoxicity (Kim et al., 2007).

Thyroid hormones are of crucial importance for the normal function of nearly every organ, as they are involved in normal brain development, control of metabolism, and many other substantial aspects of normal adult physiology. Therefore, adverse effects may impact development, metabolism, or adult physiology if there are changes in the function of the thyroid gland or intervention with the ability of thyroid hormone to exert its action (Brent, 2012). However, the mechanism(s) by which TBT induces toxicity have not been fully established.

Some researchers reported that the organotin can induce oxidative damage to mice cells both in vivo and in vitro (Liu et al., 2008). Oxidative stress, which results from the overproduction of reactive oxygen species (ROS), causes cellular oxidative injury such as lipid peroxidation, protein oxidation, and DNA damage (Ishihara et al., 2012).

Green tea, a popular well-known beverage worldwide, has captured considerable attention for its scientifically beneficial effects on human health. Most of its significant effects are attributed to its polyphenolic flavonoids, known as catechins (epicatechin, epigallocatechin, epicatechin-3-gallate, as well as the major flavonoid (−)-epigallocatechin-3-gallate). The most pervasive recognized properties of green tea are their antioxidant activities, arising from their ability to scavenge reactive oxygen species (Rietveld & Wiseman, 2003; Clement, 2009).

Taken the above-mentioned considerations, the present study is conducted specifically, to investigate the probable toxic effect of Tributyltin chloride on thyroid follicular cells of adult male albino rats, and the possible impact of reactive oxygen species (ROS) on the normal function of the thyroid gland, revealing its relation to TBT toxicity. Meanwhile, to assess whether the green tea extract in low dose, simultaneously given with TBT, exerts some protective effects on the thyroid tissues.

## Material and methods

### Chemicals

Tributyltin chloride and corn oil were purchased from Sigma–Aldrich Chemical Company, St. Louis, MO, USA. Purity of TBT was 96%.

Green tea extract (GTE) was supplied in the form of tablets (200 mg) obtained from the Technomed Groups Company, Egypt; then dissolved in distilled water.

### Animals and experimental design

Forty-five specifically pathogen free adult male albino rats (weighting 200–230 g) were included in the present work. All procedures followed the guidelines for the care and handling of animals and the study protocol was approved by the ethical committee of Alexandria Faculty of Medicine.

The animals were housed under the same laboratory conditions of light and temperature with free access to standard laboratory food and water. They were acclimatized to the new circumstances for one week prior to the start of the experiment. The duration of the experiment was 30 days.

The rats were randomly assigned to three numerically equal experimental groups (15 animals each) as follows:

**Group I (the control group)**: which was further, divided into 3 equal subgroups, (5 rats each):

- Group Ia:
  Each rat in this group received 3 ml of distilled water orally via orogastric-tube.
- Group Ib:
  Each rat in this group received 0.4 ml of corn oil orally via orogastric-tube.
- Group Ic:
  Rats of this group received green tea extract of 150 mg/kg. body weight dissolved in 3 ml of distilled water orally via orogastric-tube (Hamdy et al., 2012).

**Group II (Tributyltin-treated group)**: each rat in this group received Tributyltin chloride dissolved in corn oil in a dose of 5 mg/kg for 30 days (Mitra et al., 2014).

**Group III (TBT + GTE group):** received Tributyltin chloride dissolved in corn oil in a dose of 5 mg/kg with concomitant administration of green tea extract (150 mg/kg body weight) for 30 days (Hamdy et al., 2012).

### Methods

#### Hormonal analysis

##### Determination of serum T3, T4 and TSH

At the end of the experimental period (30 days), blood samples were collected from rats’ carotid arteries. Centrifugation was done at 400 x g (times gravity) for 5 min and serum was kept at −20 °C for further analysis of triiodothyronine (T3), thyroxine (T4) and thyroid stimulating hormone (TSH) levels by ELISA (Kuriyama et al., 2007).

### Antioxidant enzymes and lipid peroxidation

For determination of markers of oxidative stress, blood samples were collected, without using any anticoagulant and then the blood was allowed to clot for 30 min at 25 °C. The blood was centrifuged at 700 x g for 10 min. A pipette was used to separate the top yellow serum layer without disturbing the white Buffy layer.

### Estimation of reduced glutathione (GSH)

Reduced glutathione (GSH) content was assayed by the method reported by Miwa et al., (Miwa et al., 1989) GSH determination is based on the development of a yellow color when 5,5′ dithiobis (2-nitro benzoic acid) (DTNB) is added to compounds containing sulfhydryl groups. The values are expressed as mg/dL.

### Determination of Maliondialdehyde (MDA)

The serum was added to 8.1% sodium dodecyl sulphate (SDS), 20% acetic acid solution adjusted to PH 3.5, and 0.8% thiobarbituricacid (TBA). Distilled water was added to the mixture to make a solution of 4 ml, and then subjected to heating in a water bath at 95 °C for 1 h. After cooling, 1 ml distilled water and 5 ml of n-butanol-pyridine (15:1; *v*/v) mixture were added. The absorbance of the organic layer was measured at 532 nm. A standard curve was constructed using different concentrations of 1, 1, 3, 3- Tetra methoxypropane in ethanol. The concentration of MDA in sample was determined in nmol/ml (Ohkawa et al., 1979).

### Histological study

All the rats were sacrificed by decapitation under light ether anesthesia. The thyroid glands were immediately removed from animals at the time of sacrifice, and the tissues were processed for light and electron microscopic examination.

- Light microscopic study
  One lobe of the thyroid gland was fixed in 10% of neutral buffered formalin, dehydrated and embedded in paraffin. Then the 5-μm-thickness paraffin sections were cut and stained with hematoxylin and eosin (H&E) stains (Bancroft & Gamble, 2008).
- Electron microscopic study
  Specimens for electron microscopic examination were immediately fixed in 2.5% glutaraldehyde buffered with 0.1 M phosphate buffer at pH 7.4 and then post-fixed in 1% osmium tetroxide in the same buffer. They were dehydrated and then embedded in epoxy resin. Ultrathin sections were cut, double stained with uranyl acetate and lead citrate and examined with a JEOL 100 CX electron microscope (JEOL, Tokyo, Japan) at Electron Microscopic Unit, Faculty of Science, Alexandria University (Hayat, 2000).
- Histomorphometric study:
  The colloid area of 10 randomly selected thyroid follicles were measured from the H&E-stained sections of the thyroid gland of each animal per group at magnification of 100 using the Image Analyzer (Olympus BX41TF, Tokyo, Japan) at the Cell Biology Department, Medical Research Institute, Alexandria University. Such data were subjected to bio statistical analysis.

### Statistical analysis

Analysis of data was done using IBM SPSS software package version 20 and the following were calculated: Range (minimum and maximum), mean and standard deviations. For normally distributed data, comparison between the three studied groups was analyzed using F-test (ANOVA) and Post Hoc test (LSD). *p*-value less than 0.05 was considered statistically significant.

## Results

### Biochemical results

In the present work, no statistical difference was found among the three subgroups of the control group. A significant decrease in the levels of T3 and T4 has been recorded after 30 days of TBT administration when compared with both the control and the TBT+ GTE groups (Tables 1 and 2). On the contrary, the serum level of TSH was significantly increased in the TBT treated group (Table 3). In addition, the level of serum GSH was decreased in the TBT treated group (Tables 4). Table 5 demonstrates a significant increase in serum MDA level in the TBT treated group when compared with the other two groups.

**Table 1:** Serum level of T3 in the TBT-treated group compared with the control and the TBT + GTE groups

T3 (ng/dl)	Control group (*n* = 15)	TBT treated group (*n* = 15)	TBT + GTE group (*n* = 15)	F	p
Min. – Max.	79.0–108.0	39.0–49.0	53.0–60.0	351.426^*^	<0.001^*^
Mean ± SD.	93.13 ± 8.22	43.13 ± 3.40	57.20 ± 2.46		
Median	92.0	42.0	57.0		
Significance between groups	p_1_ < 0.001^*^, p_2_ < 0.001^*^, p_3_ < 0.001^*^				

F: F test (ANOVA), Significance between groups was done using Post Hoc Test (LSD)

p_1_: *p* value for comparing between Control and TBT treated groups

p_2_: *p* value for comparing between Control and TBT + GTE groups

p_3_: *p* value for comparing between TBT treated and TBT + GTE groups

*: Statistically significant at *p* ≤ 0.05

**Table 2:** Serum level of T4 in the TBT-treated group compared with the control and the TBT + GTE groups

T4 (μg/dl)	Control group (*n* = 15)	TBT treated group (*n* = 15)	TBT + GTE group (*n* = 15)	F	p
Min. – Max.	4.30–5.50	1.20–2.30	1.99–2.40	385.726^*^	<0.001^*^
Mean ± SD.	4.80 ± 0.43	1.57 ± 0.38	2.16 ± 0.15		
Median	4.80	1.50	2.20		
Significance between groups	p_1_ < 0.001^*^, p_2_ < 0.001^*^, p_3_ < 0.001^*^				

F: F test (ANOVA), Significance between groups was done using Post Hoc Test (LSD)

p_1_: *p* value for comparing between Control and TBT treated groups

p_2_: *p* value for comparing between Control and TBT + GTE groups

p_3_: *p* value for comparing between TBT treated and TBT + GTE groups

*: Statistically significant at *p* ≤ 0.05

**Table 3:** Serum level of TSH in the TBT-treated group compared with the control and the TBT + GTE groups

TSH (μIU/ml)	Control group (*n* = 15)	TBT treated group (*n* = 15)	TBT + GTE group (*n* = 15)	F	p
Min. – Max.	0.11–0.39	2.58–3.18	0.18–2.73	130.483^*^	<0.001^*^
Mean ± SD.	0.28 ± 0.09	2.81 ± 0.20	1.65 ± 0.71		
Median	0.31	2.77	1.76		
Significance between groups	p_1_ < 0.001^*^, p_2_ < 0.001^*^, p_3_ < 0.001^*^				

F: F test (ANOVA), Significance between groups was done using Post Hoc Test (LSD)

p_1_: *p* value for comparing between Control and TBT treated groups

p_2_: *p* value for comparing between Control and TBT + GTE groups

p_3_: *p* value for comparing between TBT treated and TBT + GTEgroups

*: Statistically significant at *p* ≤ 0.05

**Table 4:** Comparison between the studied groups according to the level of reduced glutathione

Reduced glutathione (mg/dl)	Control group (*n* = 15)	TBT treated group (*n* = 15)	TBT + GTE group (*n* = 15)	F	p
Min. – Max.	1.32–1.61	1.09–1.21	1.11–1.23	142.538^*^	<0.001^*^
Mean ± SD.	1.46 ± 0.08	1.14 ± 0.04	1.19 ± 0.04		
Median	1.47	1.12	1.20		
Significance between groups	p_1_ < 0.001^*^, p_2_ < 0.001^*^, p_3_ = 0.023^*^				

F: F test (ANOVA), Significance between groups was done using Post Hoc Test (LSD)

p_1_: *p* value for comparing between Control and TBT treated groups

p_2_: *p* value for comparing between Control and TBT + GTE groups

p_3_: *p* value for comparing between TBT treated and TBT + GTEgroups

*: Statistically significant at *p* ≤ 0.05

**Table 5:** Comparison between the studied groups according to the level of MDA

MDA (nmol/ml)	Control group (*n* = 15)	TBT treated group (*n* = 15)	TBT + GTE group (*n* = 15)	F	p
Min. – Max.	11.60–37.80	50.30–65.20	40.40–50.40	98.915^*^	<0.001^*^
Mean ± SD.	29.81 ± 7.19	57.57 ± 5.28	46.20 ± 3.02		
Median	32.70	55.70	47.10		
Significance between groups	p_1_ < 0.001^*^, p_2_ < 0.001^*^, p_3_ < 0.001^*^				

F: F test (ANOVA), Significance between groups was done using Post Hoc Test (LSD)

p_1_: *p* value for comparing between Control and TBT treated groups

p_2_: *p* value for comparing between Control and TBT + GTEgroups

p_3_: *p* value for comparing between TBT treated and TBT + GTEgroups

* Statistically significant at *p* ≤ 0.05

### Histological results

#### Histomorphometric study:

The mean colloid area of the thyroid follicles was significantly lowered in TBT treated group relative to the control group. On the other hand, the mean colloid area of the third group did not show any significant change when compared with the control group (Table 6).

**Table 6:** Comparison between the different studied groups according to colloid area of thyroid follicles in pixels

Area × 10^3^	Control group (*n* = 15)	TBT treated group (*n* = 15)	TBT + GTE group (*n* = 15)
Min. – Max.	13.5–160.9	7.1–24.3	6.06–160.7
Mean ± SD.	80.1 ± 50.2	15.4 ± 4.3	73 ± 49.8
Median	80.5	15.1	72.3
Significance between groups	p_1_ = 0.012^*^, p_2_ = 0.8430, p_3_ = .023^*^.		

Significance between groups was done using Post Hoc Test (LSD)

p_1_: *p* value for comparing between Control and TBT treated groups

p_2_: *p* value for comparing between Control and TBT + GTE groups

p_3_: *p* value for comparing between TBT treated and TBT + GTE groups

*: Statistically significant at *p* ≤ 0.05

### Light microscopic results:

#### Group I (control group):

**Fig. 1:**
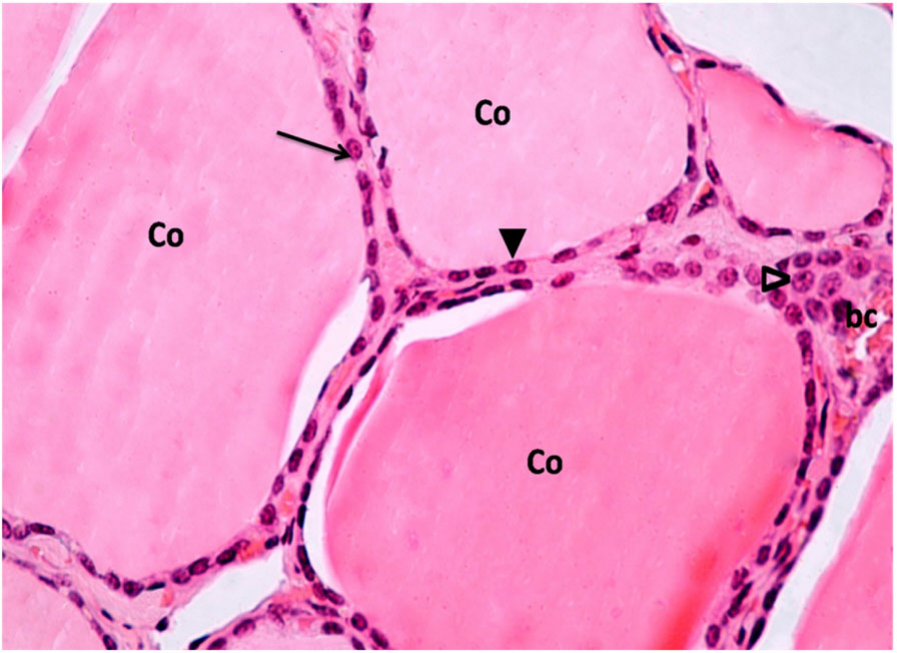
A Photomicrograph of a section in thyroid gland of a control rat (group I). It is showing multiple follicles filled with homogenous acidophilic colloid (Co). The follicles are lined with flattened to cuboidal follicular cells with oval (▲) to rounded (↑) pale nuclei. The interfollicular cells (∆) and a part of a blood capillary (bc) can also be seen inbetween the follicles. H&E stain Mic.Mag. X 400

Light microscopic examination of sections of thyroid gland showed; multiple follicles filled with acidophilic homogenous colloid. The lining follicular cells were flattened to cuboidal in shape with oval to rounded pale nuclei Fig. 1.

#### Group II (Tributyltin treated group):

**Fig. 2:**
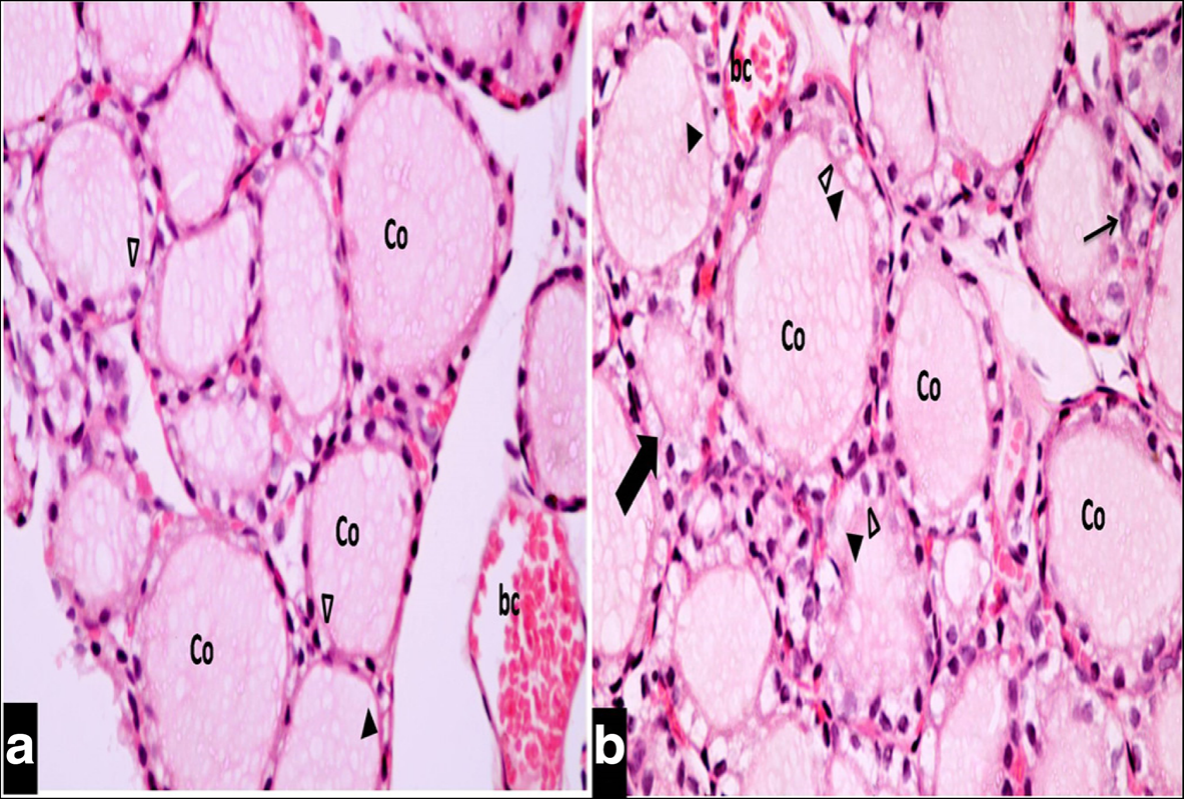
**a** & **b**: A Photomicrograph of a section in thyroid gland of a TBT-treated rat (group II). It reveals extensive vacuolated colloid (Co) filling the follicular lumen. Most of the follicular cells appear swollen and vacuolated (∆), while others show loss of their nuclei (▲). The other follicle is lined by multiple layers of follicular cells (↑). A small follicle with narrow lumen (thick ↑) can also be seen. A nearby congested blood capillary (bc) is observed. H&E stain. Mic.Mag. X 400

**Fig. 3:**
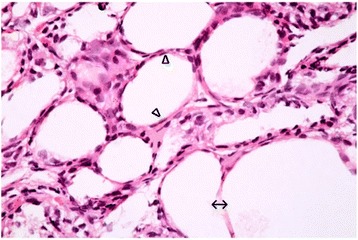
A Photomicrograph of a section in thyroid gland of a TBT-treated rat (group II). It is showing disruption of the normal architecture of the gland. The follicular lumina are empty. Some of the follicles are fused (↔). Most of the follicular cells appear flattened with dark flattened nuclei (∆). H&E stain. Mic.Mag. X 400

Examination of sections of thyroid gland of TBT-treated rat revealed markedly vacuolated colloid. Most of the follicular cells appeared swollen and vacuolated. Other cells showed loss of their nuclei. Congested blood capillaries were also observed. Figure 2a & b Stratification of the epithelial lining was encountered in some follicles. Small sized follicles were also depicted. Figure 2b More disruption in the architecture of the glands was noticed, manifested by empty fused follicles lined by flattened cells with dark flattened nuclei Fig. 3.

#### Group III (TBT+ GTE group):

**Fig. 4:**
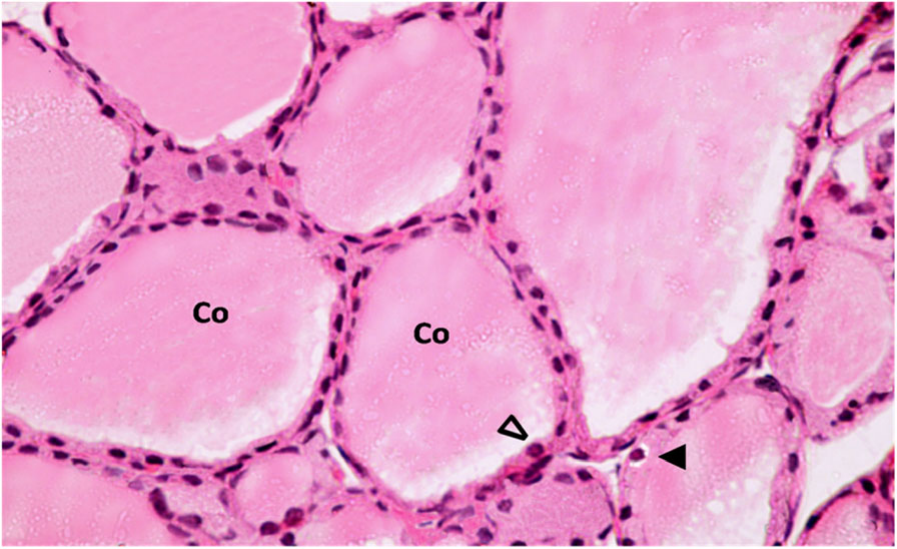
A Photomicrograph of a section in thyroid gland of a TBT-treated rat that received green tea (group III). It is showing moderately vacuolated colloid (Co) filling the follicular lumen. Most of the follicular cells appear with oval to rounded nuclei (∆) and normal cytoplasm, while others still reveal mild vacuolated cytoplasm (▲). H&E stain Mic.Mag. X 400

Light microscopic examination of sections of thyroid gland of this group revealed; preservation of the architecture of the gland. Moderately vacuolated homogenous acidophilic colloid filling the lumina of the follicles. The lining cells showed normal appearance of the nucleus and the cytoplasm. On the other hand, few vacuolated cells were still encountered Fig. 4.

### Electron microscopic results:

#### Group I (control group):

**Fig. 5:**
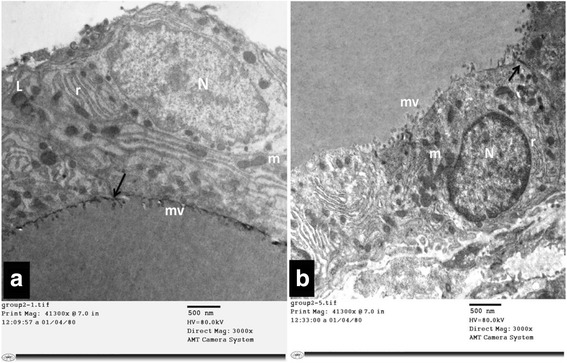
**a** & **b**: Electron micrographs of thyroid follicular cells of control rat (Group I). It is showing flattened to cuboidal cells with oval to rounded euchromatic nucleus (N) and microvillous border (mv). The cytoplasm reveals parallel arrays of rough endoplasmic reticulum (r), mitochondria (m) and lysosomes (L). An intact tight junction (↑) is also noticed

Ultrastructural examination of thyroid follicular cells of the control group (Group I) showed a flattened to cuboidal cell with a microvillous border and an oval to rounded euchromatic nucleus. The cytoplasm revealed mitochondria, rough endoplasmic reticulum and lysosomes. An intact tight junction was also encountered Fig. 5a & b **.**

#### Group II (Tributyltin-treated group)

**Fig. 6:**
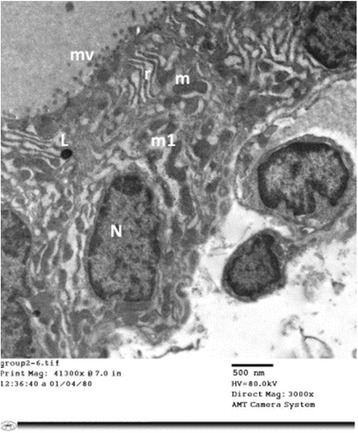
An electron micrograph of thyroid follicular cells of TBT-treated rat (Group II). It is showing high columnar cells with oval nuclei (N). The cytoplasm reveals multiple dilated profiles of rough endoplasmic reticulum (r). Some of the mitochondria appear normal (m) while other exhibit disrupted cristae (m1). Notice: (mv); microvilli, (L); Lysosomes

**Fig. 7:**
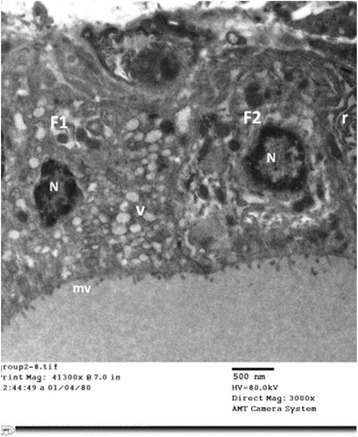
An electron micrograph of thyroid follicular cells of TBT-treated rat (Group II). It is showing two cuboidal cells (F1&F2). The microvilli (mv) are short and blunt. The nuclei (N) are small and irregular with peripheral clumping of heterochromatin. F1 cell exhibits numerous vesicles (V). F2 cell depicts dilated profiles of rough endoplasmic reticulum (r)

**Fig. 8:**
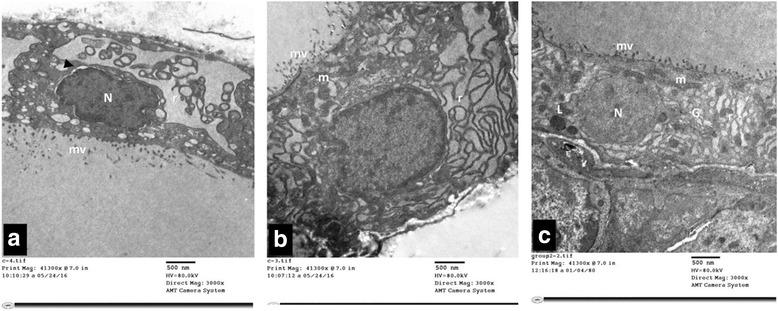
**a**, **b** & **c**: Electron micrographs of thyroid follicular cells of TBT-treated rat (Group II). It reveals cuboidal follicular cells with microvillous border (mv). The cytoplasm is filled with extensively dilated rough endoplasmic reticulum (r) that filled with flocculent material. Photo **a** shows irregular nucleus (N) with dilated perinuclear cisterna (▲). Photo **b** depicts mitochondria (m) with disrupted cristae. A large Golgi complex (G) is encountered in photo (**c**). Notice: (L); lysosomes

**Fig. 9:**
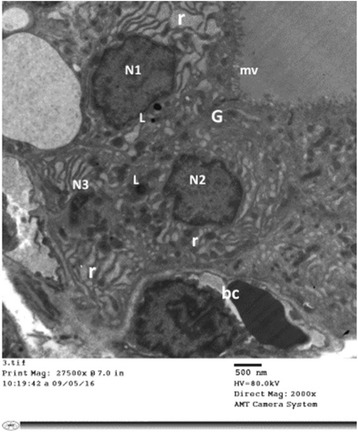
An electron micrograph of thyroid follicular cells of TBT-treated rat (Group II). It is showing follicular cells arranged in layers. The nuclei (N1&N2) are euchromatic, while N3 is small and heterochromatic. The cytoplasm reveals dilated rough endoplasmic reticulum (r) and multiple lysosomes (L). Notice: (G); Golgi apparatus, (mv); microvilli, (bc); blood capillaries

Electron microscopic examination of thyroid follicular cells of TBT-treated rats (Group II) revealed cuboidal to high columnar cells Fig. 6. Short, blunt and disrupted microvilli were depicted in some cells Fig. 7. The nuclei of some cells were normal in shape and euchromatic while other cells showed changes varied from small irregular and dark nuclei Figs. 7 and 9 to dilatation of perinuclear cisterna Fig. 8a. The cytoplasm showed mild to markedly dilated rough endoplasmic reticulum Figs. 6, 7, 8 and 9. The lumina of some of them were filled with flocculent material Fig. 8a & b. Mitochondria with disrupted cristae were also encountered Figs. 6 and 8b **.** Numerous vesicles and lysosomes were also noticed Figs. 7 and 9. Some of the follicular cells were arranged in layers Fig. 9.

#### Group III (TBT + GTE group)

**Fig. 10:**
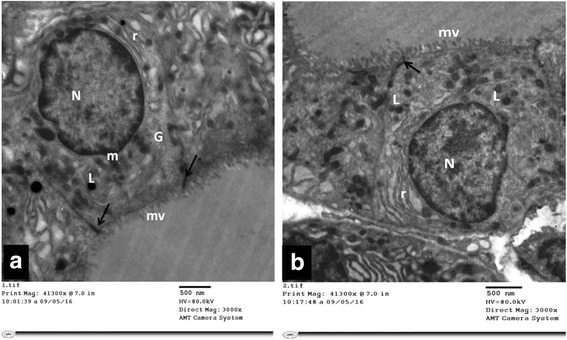
**a** & **b**: Electron micrographs of thyroid follicular cells of TBT + GTE group (Group III). It is showing cuboidal cells with a well-developed microvillous (mv) border. The nucleus (N) is euchromatic and rounded. The cytoplasm shows normal to mildly dilated rough endoplasmic reticulum (r), lysosomes (L), mitochondria (m) and Golgi apparatus (G). An intact tight junction (↑) is also noticed

Ultrastructural examination of thyroid follicular cells of this group that received TBT + GTE (Group III) showed marked improvement of most of the examined follicular cells. They were cuboidal in shape and exhibited a well-developed microvillous border. Their nuclei were euchromatic and rounded. The cytoplasm showed normal profiles of rough endoplasmic reticulum Fig. 10a. Although mildly dilated rough endoplasmic reticulum and multiple lysosomes were still encountered in some cells Fig. 10b **.**

## Discussion

Over the last decades, a dramatic increase in hormonal disorders was reported, and it has been assumed that growing exposure to endocrine disrupting chemicals (EDCs) contributes to the burden of endocrine disorders among populations. However, a deluge of research in the field of endocrine disruption has focused on estrogenicity (Sikka & Wang, 2008; Roy et al., 2009).

At present, there are scanty reports on the impact of TBT on thyroid tissues. Thereby, this spurred us to conduct an oral toxicity study to provide further inclusive information pertaining to the effect of Tributyltin on the thyroid follicular cells.

Thyroid hormone homeostasis appears to be the target of plentiful environmental chemicals either natural or manufactured. In mammals, thyroid glandular activity is commonly determined by thyroid hormone secretion rate (Zoeller, 2007). Taking into consideration that the structure of any organ closely reflects the state of its function; histological examination of the thyroid gland provides a sensitive early indicator of the glandular activity than serum T3 and T4 levels.

In this context, the primary aim of the present study was to assess the alteration in thyroid homeostasis that might occur following exposure to Tributyltin. Furthermore, this study aimed to identify potential associations between TBT and reactive oxygen species (ROS) on the normal function of the thyroid gland and the probable protective role of green tea extract when given simultaneously with TBT.

The dosing regimen and the duration selected in the current study was in accordance with the work of (Mitra et al., 2014) who studied the sub chronic toxicity of TBT in rats. He reported that one month exposure to TBT in low doses resulted in loss of cell viability in liver, kidney as well as the lungs.

The male rats were selected in the current study, as increasing evidence elucidates that estrogen influence the risk of thyroid diseases. It also plays a crucial role as a promoting factor in thyroid tumorigenesis (Derwahl & Nicula, 2014).

In the present study, the rats treated with Tributyltin demonstrated significant decrease in the serum levels of T3 and T4 along with the evident increase in TSH levels as compared with the control group. Similar disruption of the levels of these hormones were reported by previous researchers (Adeeko et al., 2003; Wang et al., 2008; Sharan et al., 2014) who attributed its occurrence to the antithyroid effect of TBT.

This was further asserted by histological examination of follicular cells, which unveiled evident structural changes, reflecting augmented activity in thyroid follicles of this group, in response to hypersecretion of TSH to compensate the decreasing levels of T3 and T4. These changes were manifested by swollen vacuolated follicular cells, epithelial stratification, along with excessive vacuolation of the colloid. Congested blood vessels were also encountered. These changes were further bolstered by morphometric and statistical analyses that revealed a significant decrease in the mean colloid area of thyroid follicles as compared with their respective controls.

In accordance with our results, (Pereira et al., 2013) reported disorganization of follicular cell groups, with hypertrophy, hyperplasia of thyrocytes and glandular congestion compared with control thyroid gland. On the other hand, he found no changes in plasma levels of T_3_ and T_4_ after 15 days of treatment in his study. This could be clarified by the shorter duration of his research when compared with the present one.

It is acknowledged that prolonged stimulation of the pituitary by decreasing levels of thyroid hormones results in release of elevated levels of TSH by the thyrotrophs which may lead to thyroid gland neoplasia manifested as shrinkage of colloid area, hypertrophy as well as hyperplasia of follicular cells (Hood et al., 1999; Boelaert, 2009). Moreover, the present study demonstrated small sized follicles, empty and fused follicles accompanied by disturbance of the normal architecture of the gland. Similar findings were reported by (Wang et al., 2008) while studying the effect of TBT on thyroid gland of *Xenopus laevis*. They stated that TBT can induce intense damage to the thyroid tissues. This damage manifested by reduction in follicular region, colloid depletion and malformed follicles.

Ultra-structurally, the follicular cells revealed mild to moderately dilated rough endoplasmic reticulum. Mitochondria with disrupted cristae were also encountered together with ample lysosomes and vesicles. Some follicular cells showed cuboidal to high columnar cells. Meanwhile, some of the nuclei appeared small and shrunken with peripheral clumping of heterochromatin, while other nuclei showed dilated perinuclear cisternae.

Our results are consistent with those of (Sharan et al., 2014) who studied the effect of TBT on thyroid gland, they declared that TBT possessed antithyroid effect via intervention with thyroid hormone regulation. Such disturbance occurred through decreasing the transcription of thyroid hormone receptors (TR) by disrupting the physiological concentrations of thyroid hormones, thereby increasing the ligand-dependent cooperativity of TR with the co-repressors and shedding of the co-activator. Additionally, TBT caused down-regulation of the thyroid peroxidase and thyroglobulin genes, which correlated with the decrease in T3 and T4, while boosting the thyroid stimulating hormone (TSH) levels. Furthermore, Tributyltin can cause up-regulation of thyroid-stimulating hormone receptor in the thyroid glands.

However, it is quite apparently that thyroid gland activity is positively regulated by thyroid stimulating hormone (TSH) synthesized and secreted from pituitary thyrotrophs, whose activity is in turn controlled by the hypothalamic TSH-releasing hormone (TRH). TSH acts on specific receptors on the membrane of follicular cells and invigorates the activity of the sodium-iodine symporter and of intracellular enzymes involved in thyroid hormone synthesis.

Therefore, when the level of serum thyroid hormone dwindles, the feedback inhibition of TSH is attenuated and more TSH is secreted; this promotes thyroid cell hyperplasia and hypertrophy and stirs the function of the thyroid into the active state to sustain the body thyroid hormone needed (Chiamolera & Wondisford, 2009).

In the context, (Scanlan et al., 2004) have speculated that increased lysosomal activity in the follicular cells, is simply a reflection of augmented cellular secretory activity initiated by high levels of circulating TSH. It could also be triggered by enhanced phagocytosis secondary to the degenerative changes noticed in some cells.

Myriads of reports (Patrick, 2009; Jugan et al., 2010) suggest that thyroid disruptors can target the thyroid endocrine cascade at various levels encompassing several molecular components of the hypothalamus–pituitary–thyroid-periphery (HPTP) axis as well as the functioning of the peripheral tissues including; iodine uptake, thyroid hormone production, interconversion of thyroid hormones, cellular uptake and cell receptor activation.

Thyroid follicles represent the functional subunit of thyroid tissue. Each follicle is lined by a single epithelial cell layer and is filled with a thyroglobulin (TG) containing colloidal mass formed in the rER and serving as the matrix for thyroid hormone (TH) synthesis.

In addition, TG also plays an imperative role in modulating the expression of genes involved in the synthesis of other thyroid proteins (sodium-iodide symporter [NIS] and thyroid peroxidase [TPO]) and transcription factors involved in normal thyroid physiology (Sellitti et al., 2001).

Glycosylation of TG begins in the rough endoplasmic reticulum (rER) and is completed in the Golgi complex. Within thyroid follicles, newly synthesized TG is transported along the secretory route to the apical plasma membrane of thyroid epithelial cells. After exocytosis, TG is stored within the extracellular lumen of thyroid follicles in a covalently cross-linked form (van de Graaf et al., 2001).

Because TH are iodothyronine derivatives, uptake of iodide from the blood stream represents a substantial step in their biosynthesis. During the process of generating TH, tyrosine residues on the TG molecule are coupled with iodine at the apical pole of thyrocyte. This iodination process is called organification and is controlled by the enzyme thyroid peroxidase (TPO). Thus, monoiodotyrosine (MIT) and diiodotyrosine (DIT) are formed. The coupling of MIT and DIT is also mediated by TPO as well as linking two DIT molecules to form T4. In addition to thyroglobulin and iodide, TPO requires H_2_O_2_ as a third factor to carry out the above-mentioned reactions. H_2_O_2_ production is presumably a rate-limiting step during TH generation (Song et al., 2007).

At the thyrocyte level, stimulation of the TSH receptor (TSHR) by TSH instigates several second messenger signaling cascades leading to increased iodide uptake, TH synthesis and secretion (Roger et al., 2010; Vassart & Costagliola, 2011).

It is well known that the morpho functional status of each follicle is controlled not solely by the TSH level, but rather by other factors including thyroglobulin contained within the follicle (Suzuki et al., 2011).

Thyroid hormone liberation begins with endocyto**s**is of small amounts of colloid into vesicles that are transported inside the follicular cells. Lysosomes then fuse with these vesicles and release T4/T3. Each TG molecule stores ten times more T4 than T3 (Scanlan et al., 2004).

Although the damage of follicular cells in the thyroid seems to be a reason for impaired thyroid hormones, yet a prospective role of oxidative stress might be another reason.

The further step of the study was to evaluate the susceptibility of reactive oxygen species (ROS) to contribute in the modulation of thyroid structure.

Under normal physiological conditions, reductive power of a cell is achieved by the amount of reduced glutathione. It helps to attain oxidative damage under control by various processes and reduction in GSH leads to stress (Lushchak, 2012).

Malondialdehyde (MDA) is one of the major oxidation products of peroxidized polyunsaturated fatty acids, and thus increased MDA content is an imperative indicator of lipid peroxidation (Rahal et al., 2014).

The results of the present study depicted significant decrease in the level of serum GSH, as well as an increase in the level of serum MDA levels in the TBT treated group in relation to the other groups.

In concomitant with the previous results, ROS generation may be a radical factor underlying TBT toxicity. Multitude of studies (Demir et al., 2011; Mitra et al., 2013; Zhang et al., 2014; Bernat et al., 2014) have suggested that the generation of ROS, including the species derived from H_2_O_2_ such as OH·, is one of the mechanisms involved in TBT toxicity. ROS cause damage to mitochondrial and other cytoplasmic organelle membrane structures through peroxidation of phospholipids, proteins and nucleotides. Consequently, membrane stability and integrity being disrupted resulting in osmolality changes and hydropic cell degeneration (Guo et al., 2013). Meanwhile, lipid peroxidation activates endonuclease enzymes, with subsequent breakdown of nuclear DNA and nuclear degeneration (Zhang et al., 2016).

On the other hand, (Mitra et al., 2014) reported that; reactive oxygen species but not lipid peroxidation content was observed to be significantly elevated both in the tissues and serum after a month of low dose of TBT exposure.

Similarly, (Gupta et al., 2011) on his research on thymus; clarified that oxidative stress and apoptosis are two inseparable phenomena in TBT toxicity.

As a recap, it is obvious that oxidative stress as the final manifestation of a multi-step pathway, refers to cellular status imbalance between the ROS level and the cellular antioxidant defense system due to the depletion of antioxidants, or the excessive accumulation of ROS, or both, which leads to cellular damage. It has been demonstrated that exposure to TBT could yield ROS which cause various organ lesions. Approximately 0.1% of all oxygen entering the mitochondrial electron transport chain is released as ROS, which can disrupt intracellular redox status and result in homeostasis disorder (Zhang et al., 2008; Zhou et al., 2010).

Based upon the work done by (Li et al., 2015) a significant elevation of the oxidative stress indices was observed following TBT exposure, which suggested that oxidative stress was induced by TBT. Upon amalgamating previous results with the findings of this study, it is deduced that oxidative damage is one of the critical toxic mechanism of TBT.

Furthermore, (Sugawara et al., 2002) stated that reactive oxygen species can also inactivate thyroid peroxidase enzyme via two mechanisms, reversible formation of compound III due to excessive H_2_O_2_ or O_2_^−^, and irreversible free radical-mediated thyroid peroxidase (TPO) inactivation through attacking the active site of thyroid peroxidase enzyme, causing inactivation of the catalytic site of this enzyme resulting in disruption of the level of thyroid hormones.

In the current work, co administration of green tea extract (GTE) with TBT for 30 days resulted in considerable thyroid preservation. Few cells still showed mildly dilated rER and numerous lysosomes. These histological effects correlated as well with the morphometric and biochemical results that showed significant amelioration of these attributes.

These data were on a par with (Liu et al., 2008) who reported that green tea polyphenols (GTPP) were effective in reducing TBT-induced oxidative damage both in vivo and in vitro. They found that (ROS) production and malondialdehyde content of the liver in mice exposed to TBT were dwindled in the GTPP-treated group compared to the untreated group. Moreover, they demonstrated that the number of cells with damaged DNA in untreated mice was figured out to be significantly higher compared to GTPP-treated mice. Furthermore, damage to the nuclei and mitochondria observed in TBT-treated mice were alleviated in mice treated with both TBT and GTPP. They attributed this protective role to the powerful ability of GTPP to scavenge ROS and hinder DNA breaks.

Several studies (Narotzki et al., 2012; Giménez et al., 2013) reported that green GTE constitutes an essential source of antioxidants. Besides polyphenols, GTE contains additional antioxidants such as carotenoids, tocopherols (vitamin E derivatives) and vitamin C. Tea contains further minerals that function as co-factors in antioxidant enzymes: zinc, selenium and manganese. Polyphenols have supplementary mechanisms in which they reduce oxidation level besides direct role as antioxidants.

As antioxidants, green tea polyphenols either chelate redox-active metal ions, such as iron and copper preventing the formation of metal-catalyzed free radicals or scavenge reactive oxygen/nitrogen species or modulate antioxidant enzymes (Nakagawa & Yokozawa, 2002).

On the contrary, prior researches (Chandra et al., 2011; Abulfadle et al., 2015) have declared that excessive and high doses of green tea have the potential to alter the thyroid gland physiology and architecture, that is, enlargement of thyroid gland as well as hypertrophy and/or hyperplasia of the thyroid follicles and inhibition of the activity of thyroid peroxidase and 50-deiodinase I with elevated thyroidal sodium-potassium- ATPase activity along with significant decrease in serum T3 and T4, and a parallel increase in serum thyroid stimulating hormone (TSH).

## Conclusion

Thus, the current study provides novel information that accentuate the hazardous risk of TBT on the thyroid. Co-administration of green tea extract (natural antioxidant) improves Tributyltin induced morphological changes in thyroid architecture as well as restoration of glutathione activity.

The available toxicity data might provide a useful platform for further studies to clarify the human risk and to boost the global awareness about endocrine-disrupting chemicals.

These issues highlight the critical need to develop swift and robust tools to identify TBT compounds, as they are deeply interwoven with our daily lives from various sources. Thus, a holistic and comprehensive understanding of the mechanisms underlying thyroid disruption, may lead to changes in public policy and awareness, and make it possible to limit adverse outcomes for future generations.

## Abbreviations

DIT: Diiodotyrosine
EDCs: Endocrine disrupting chemicals
GSH: Estimation of Reduced Glutathione
GTE: Green tea extract
GTPP: Green tea polyphenols
H&E: Hematoxylin and eosin
HPTP: Hypothalamus–pituitary–thyroid-periphery
MDA: Maliondialdehyde
MIT: Monoiodotyrosine
NIS: Sodium-iodide symporter
OT: Organotin
rER: Rough endoplasmic reticulum
ROS: Reactive oxygen species
T3: Triiodothyronine
T4: Thyroxine
TBT: Tributyltin
TG: Thyroglobulin
TPO: Thyroid peroxidase
TPT: Triphenyltin
TR: Thyroid hormone receptors
TRH: TSH-releasing hormone
TSH: Thyroid stimulating hormone
TSHR: TSH receptor
WHO: World Health Organization
